# Immune-excluded and immune-suppressive tumor microenvironments: mechanisms, spatial biomarkers, and therapeutic rewiring

**DOI:** 10.3389/fonc.2026.1855429

**Published:** 2026-05-22

**Authors:** Bo Wang, Haixin Ding, Yutong Li, Xue Zhao, Pengling Ge

**Affiliations:** Heilongjiang University of Chinese Medicine, Harbin, China

**Keywords:** cancer-associated fibroblasts, immune checkpoint blockade, immune exclusion, immune suppression, immunotherapy resistance, myeloid cells, spatial biomarkers, spatial transcriptomics

## Abstract

**Background:**

Immune checkpoint inhibitors have improved outcomes in several malignancies, yet durable benefit remains limited in most solid tumors because many lesions exhibit immune-excluded or immune-suppressive tumor microenvironments (TMEs). These resistant states are now understood to arise from coordinated stromal, myeloid, cytokine, vascular, and metabolic programs that prevent effective antitumor immunity and sustain therapeutic failure.

**Methods:**

This review synthesizes recent evidence on the biological architecture of immune-excluded and immune-suppressive TMEs, with particular emphasis on stromal remodeling, cancer-associated fibroblasts, myeloid-cell dominance, cytokine and chemokine networks, vascular dysfunction, metabolic stress, and organ-specific niche effects. We further integrate emerging data from spatial transcriptomics, multiplex imaging, spatial proteomics, and related platforms to evaluate how spatial biomarkers may refine patient stratification and therapeutic decision-making.

**Results:**

Current evidence indicates that resistant TMEs are spatially organized and dynamically evolving ecosystems rather than static histologic phenotypes. CAF/ECM remodeling, suppressive myeloid populations, cytokine circuits, vascular dysfunction, and metabolic stress cooperate to impair T-cell trafficking, infiltration, and effector fitness. Spatially resolved technologies may help refine patient stratification by identifying dominant resistance modules, although prospective clinical validation remains limited.

**Conclusion:**

Immune-excluded and immune-suppressive TMEs represent actionable but heterogeneous resistance states. Future progress will depend on integrating spatially informed biomarker systems, longitudinal profiling, and mechanism-based combination therapies to convert nonresponsive tumors into immunologically permissive niches.

## 1 Introduction

Immune checkpoint inhibitors (ICIs) have reshaped the treatment landscape of several malignancies, yet durable benefit remains confined to a minority of patients across most solid tumors (1). A major reason is that therapeutic response is not determined solely by tumor-intrinsic genetics, but also by the surrounding tumor microenvironment (TME). The TME governs whether antitumor immunity can be initiated, spatially organized, and sustained. This view is consistent with earlier conceptual work showing that tumor-promoting inflammation is a core hallmark of cancer. It also aligns with the cancer-immunity cycle, in which effective immunity requires antigen release and presentation, T-cell priming, trafficking, infiltration, and cytolytic activity (2, 3). In practical terms, many tumors remain immunologically “cold”, with sparse cytotoxic lymphocyte infiltration and poor responsiveness to checkpoint blockade, whereas others display partial immune engagement but still fail to mount productive antitumor immunity because suppressive stromal and myeloid programs dominate local tissue function (1). These observations have shifted attention from tumor cells alone toward the ecological logic of the TME as a central determinant of immune escape and treatment resistance (4). However, despite substantial recent progress, many contemporary reviews have emphasized selected regulatory axes or disease-specific updates, whereas the broader integration of immune contexture, spatial immune failure, and multi-compartment resistance programs remains less explicitly synthesized in a unified framework (5, 6).

Within this broader framework, immune-excluded and immune-suppressive TMEs have emerged as especially relevant clinical and biological states. Immune exclusion refers to the failure of effector T cells to penetrate tumor nests despite their presence at the invasive margin or surrounding stroma, whereas immune suppression more broadly describes a tissue context in which infiltrating immune cells are functionally restrained by inhibitory cytokines, suppressive myeloid populations, stromal signaling, metabolic stress, or abnormal vasculature (1). This distinction matters because lack of response to immunotherapy is often not simply a question of whether immune cells are present, but where they are positioned, what barriers restrict their trafficking, and which local cues erode their effector fitness (7). Increasingly, therefore, the TME is being viewed not as a passive background but as a spatially organized and dynamically adaptive system that can either support immune surveillance or actively redirect it toward tolerance and escape. In this context, the distinction between immune-excluded and immune-suppressive states is especially useful because it links immune failure not only to the abundance of infiltrating cells, but also to their topography, functional state, and stromal constraint, consistent with the broader concept of immune contexture in human tumors (5–7). In this review, we use this distinction not merely as a descriptive classification, but as a conceptual framework for understanding how resistant TMEs are spatially assembled, biologically maintained, and potentially therapeutically rewired.

Several converging lines of evidence indicate that this resistant ecosystem is maintained by coordinated interactions among stromal, myeloid, vascular, and soluble mediators rather than by a single dominant pathway. Cancer-associated fibroblasts (CAFs) are now recognized as major architects of these niches through extracellular matrix remodeling, fibrosis, growth factor secretion, and the establishment of physical and biochemical barriers that favor immune exclusion and therapy resistance (8). In parallel, monocytes and monocyte-derived populations contribute substantially to immune tolerance, angiogenesis, and tumor dissemination, reflecting the remarkable plasticity of the myeloid compartment within tumors (9). Recent work has further highlighted reciprocal CAF–tumor-associated macrophage (TAM) signaling as a particularly important suppressive axis, while extracellular matrix stiffness and macrophage polarization appear to reinforce exclusionary tissue states that are difficult to reverse once established (10, 11). Together, these studies argue against a reductionist cell-by-cell view of the TME and instead support a niche-based model in which stromal architecture, inflammatory signaling, and immune cell programming are tightly intertwined (8, 10, 11).

A second major advance has been methodological rather than conceptual. Earlier frameworks established that immune-cell composition and localization are biologically meaningful determinants of tumor behavior, but recent spatially resolved technologies now allow these principles to be examined directly within intact tissue architecture at much higher resolution (5, 7). Compared with bulk profiling or conventional immunohistochemistry, spatial transcriptomics, multiplex imaging, and related multimodal platforms can resolve where specific cell states are positioned, which cellular neighborhoods are formed, and how regional suppressive programs shape treatment response. These approaches have opened the way for spatial biomarkers that may complement, rather than simply replace, conventional immune readouts (7). In parallel, recent translational reviews have emphasized that high-resolution TME profiling should be embedded into clinical trial design if biomarker-matched combinations are to move beyond empiricism (4).

This evolving evidence base has important therapeutic implications. Importantly, this TME-centered focus should not be interpreted as implying that all immunotherapy resistance is microenvironmental. Tumor-cell intrinsic mechanisms, including defects in antigen presentation, impaired IFN-γ/JAK–STAT signaling, antigen loss, and oncogene-driven immune evasion, can independently limit immune recognition or effector killing. We therefore discuss TME-mediated resistance as one major layer of immunotherapy failure, while explicitly distinguishing it from tumor-intrinsic resistance mechanisms where therapeutically relevant. If immune-excluded and immune-suppressive tumors are maintained by layered stromal, myeloid, cytokine, and spatial constraints, then successful treatment will likely require therapeutic rewiring rather than isolated checkpoint blockade alone. In that context, rational strategies may include stromal reprogramming, myeloid targeting, vascular and matrix normalization, and precision modulation of cytokine networks to restore T-cell priming, trafficking, and effector competence within resistant niches (11). Accordingly, this review focuses on three linked questions: how immune-excluded and immune-suppressive TMEs are mechanistically established; how spatial technologies can identify actionable biomarkers of resistant niches; and how these insights may be translated into mechanism-matched combination strategies capable of converting nonresponsive tumors into more immunologically permissive states (1, 7, 12).

Unlike prior cytokine-centered models of TME resistance, this review frames immune-resistant TMEs as multi-compartment niches shaped by stromal architecture, CAF/ECM remodeling, myeloid dominance, vascular dysfunction, metabolic stress, and cytokine circuits. We focus on how these interacting modules determine immune exclusion, immune suppression, and therapeutic vulnerability, and on how spatial biomarkers may guide mechanism-matched combinations.

## 2 Defining immune-excluded and immune-suppressive tumor microenvironments

A useful starting point for classifying tumor immune states is the distinction between hot and cold tumors. In this review, we use “hot tumors” to refer broadly to tumors enriched in activated cytotoxic lymphocytes and more likely to benefit from immune checkpoint blockade. By contrast, we use “cold tumors” as a broad umbrella descriptor for tumors with weak or ineffective antitumor immune activity and poor responsiveness to current immunotherapies. This term is useful clinically, but it is too coarse for mechanistic interpretation.

For clarity, we use more specific terms when describing resistant TME states. “Immune-excluded tumors” refer to tumors in which immune cells are present but remain spatially segregated from tumor cell-rich regions, often at the invasive margin or within stromal compartments. “Immune-suppressive tumors” refer to tumors in which immune cells may be present within or near tumor nests, but their effector function is restrained by suppressive myeloid cells, inhibitory cytokines, stromal signaling, metabolic stress, abnormal vasculature, checkpoint engagement, or related mechanisms. “Immune-desert tumors,” when discussed, refer more narrowly to tumors with very low immune-cell infiltration. These categories may overlap biologically, but they should not be used interchangeably.

Increasing evidence indicates that tumors with measurable immune-cell presence may still fail to respond because effector cells are spatially segregated from malignant nests, trapped in stromal compartments, or rendered dysfunctional after entry into the lesion. Accordingly, this review focuses on immune-excluded and immune-suppressive TMEs as two partially overlapping but mechanistically distinct resistance states (4, 13).

### 2.1 Immune-excluded TME: presence without effective access

The defining feature of an immune-excluded TME is not the absolute absence of immune cells, but their failure to establish productive contact with malignant cells (14). In these tumors, cytotoxic lymphocytes are frequently detected at the invasive margin, in the peri-tumoral stroma, or around vascular niches, while tumor nests remain relatively inaccessible (15). This pattern is clinically important because it implies that antitumor immunity has been initiated to some degree, yet is arrested before effective intratumoral execution. Such arrest may occur through several non-mutually exclusive mechanisms. These include fibroblast-driven extracellular-matrix deposition, abnormal vasculature, chemokine misdirection, defective antigen presentation, and suppressive interactions in lymphatic or stromal compartments. In other words, exclusion is best understood as a failure of trafficking, penetration, and local engagement rather than a simple lack of immunogenicity (8, 13, 15). In other words, exclusion is best understood as a failure of trafficking, penetration, and local engagement, rather than a simple lack of immunogenicity (7). Earlier mechanistic studies helped establish this view by showing that T-cell localization within tumors is strongly constrained by stromal architecture and matrix organization, such that lymphocytes may remain confined to peri-tumoral or stromal regions despite the presence of tumor antigens (6, 16).

Cancer-associated fibroblasts and the extracellular matrix are central to this phenotype. Foundational work in tumor stroma biology established that fibroblasts are active regulators of cancer progression rather than passive structural cells, while later mechanistic studies showed that matrix density, orientation, and chemokine-rich stromal barriers can directly restrict T-cell migration into tumor nests (16–18). CAFs do not merely provide structural support; they actively remodel matrix composition, increase tissue stiffness, secrete growth factors and inflammatory ligands, and create physical and biochemical barriers that favor immune exclusion and therapy resistance (8). More recent work has reinforced this view by showing that CAF-rich stromal regions can organize tumor architecture in ways that prevent lymphocyte access, while specific spatial CAF programs are increasingly linked to shortened survival and poor immunotherapy outcomes (4). In parallel, Tregs can reinforce exclusion outside as well as inside tumor nests by acting in the stroma, vasculature, and lymphatics to restrict immune-cell migration before infiltration occurs (15). Thus, immune exclusion should not be framed solely as a T-cell problem; it is more accurately a tissue-organization problem produced by coordinated stromal, vascular, and regulatory-cell barriers (4, 8, 15).

The relevance of this concept is apparent across tumor types. In microsatellite-stable colorectal cancer, for example, immunotherapy failure has been closely tied to limited T-cell entry and dysfunctional CD8-positive populations, despite the presence of potentially relevant immune subsets within the broader microenvironment (19). Similarly, small cell lung cancer often exhibits T-cell exclusion, deficient antigen-presentation machinery, and macrophage-centered suppressive programs, all of which help explain why the benefit of PD-1/PD-L1 blockade remains modest compared with other solid tumors (20). In pancreatic ductal adenocarcinoma, dense extracellular matrix, CAF-driven fibrosis, and myeloid accumulation collectively impair immune-cell infiltration and activation, making immune exclusion one of the most clinically consequential aspects of its resistant phenotype (21). These observations underscore that immune exclusion is not a niche curiosity of a single cancer type, but a recurrent mode of therapeutic failure across solid tumors.

### 2.2 Immune-suppressive TME: infiltration without effective function

If immune exclusion describes a state of restricted access, the immune-suppressive TME describes a state of restricted function. In these tumors, immune cells may be present within or near tumor nests, yet antitumor activity is blunted by inhibitory cytokines, suppressive myeloid populations, stromal signaling, checkpoint engagement, and metabolic competition (9, 13). Monocytes and monocyte-derived populations exemplify this logic well: they retain marked plasticity, but within tumors they are frequently instructed toward programs that promote immune tolerance, angiogenesis, dissemination, and resistance to therapy (22). Likewise, tumor-associated macrophages, MDSCs, and Tregs collectively create a local circuitry that suppresses cytotoxic lymphocytes and favors tumor persistence, often with substantial redundancy between pathways (13, 23). For this reason, many tumors that appear “inflamed” by simple cell counting are functionally nonresponsive when examined at higher resolution (14).

Tumor-intrinsic alterations can further reinforce this suppressive context. KRAS-altered tumors provide a representative example: beyond driving malignant growth, KRAS-associated states may impede effective T-cell infiltration, recruit suppressive cells such as MDSCs, Tregs, and CAFs, and generate immune landscapes that differ substantially according to co-mutation patterns (24). In lung cancer, the presence of Tregs, MDSCs, and tumor-associated macrophages has similarly been associated with poorly responsive microenvironments and has therefore been proposed as part of a biomarker framework for immunotherapy sensitivity (13). These data argue that an immune-suppressive TME is not defined simply by one inhibitory cell type or one checkpoint ligand. Rather, it is an emergent state in which multiple stromal, innate, and tumor-intrinsic mechanisms converge to diminish effector-cell fitness and sustain immune escape (13, 24).

Importantly, immune exclusion and immune suppression are conceptually distinct but biologically intertwined. A tumor may first exclude effector cells through stromal and vascular barriers, and then suppress the minority that succeed in entering. Conversely, a chronically suppressive milieu may secondarily reinforce exclusion by promoting matrix remodeling, aberrant angiogenesis, or chemokine patterns that divert lymphocytes away from tumor nests (4, 15). This overlap is one reason why static one-dimensional labels often fail to predict treatment outcome. From a translational perspective, it is therefore more useful to view these phenotypes as dynamic states along the cancer-immunity cycle, with barriers arising at antigen presentation, priming, trafficking, infiltration, recognition, or cytolytic execution (14). Such a view better explains why combination therapies that normalize vasculature, reprogram fibroblasts, target myeloid suppressors, or modulate cytokine networks may be necessary to move tumors from nonresponsive to responsive immune states.

### 2.3 Why spatial definition matters

The distinction between immune-excluded and immune-suppressive TMEs has gained sharper meaning with the rise of spatially resolved technologies. Spatial transcriptomics, multiplex imaging, and related platforms have made it increasingly clear that the positioning of immune cells can be as informative as their abundance. Indeed, recent reviews now place spatial architecture at the center of immunotherapy interpretation, showing that cell-cell proximity, stromal compartmentalization, and region-specific immune neighborhoods can shape therapeutic response in ways that bulk profiling cannot capture (4, 7). This shift has practical consequences: a tumor with moderate lymphocyte numbers may still behave as immune-excluded if those cells remain peri-tumoral, whereas a lesion with substantial infiltration may still behave as immune-suppressive if the infiltrate is dominated by exhausted or restrained effectors (14). For the purposes of this review, therefore, immune exclusion and immune suppression are treated not as rigid histologic labels, but as spatially and functionally defined states of failed antitumor immunity. For conceptual clarity and translational continuity, **Table 1** summarizes the major immune-resistant states discussed in this review, highlighting their defining features, spatial patterns, dominant mechanisms, and therapeutic implications.

**Table 1:** Conceptual comparison of immune-excluded, immune-suppressive, immune-desert, and mixed tumor microenvironment states.

Immune state	Core definition	Spatial pattern	Key mechanisms	Translational implication
Immune-excluded	Immune cells present but fail to enter tumor nests	T cells retained at invasive margin or stroma	CAF/ECM barriers; TGF-β; abnormal vessels; chemokine misdirection	Restore lymphocyte trafficking and tumor penetration
Immune-suppressive	Immune cells present but functionally restrained	Infiltrates coexist with suppressive niches	TAMs/MDSCs/Tregs; inhibitory cytokines; checkpoints; hypoxia/metabolic stress	Relieve suppression and restore effector function
Immune-desert	Minimal immune-cell infiltration	Sparse immune cells in tumor and stroma	Poor priming; low antigenicity; defective recruitment; immune ignorance	Initiate immune priming and recruitment
Mixed/transitional	Multiple states coexist or evolve over time	Regional or site-specific heterogeneity	Spatial heterogeneity; therapy-induced remodeling; organ-specific niches	Use spatial/longitudinal profiling and adaptive combinations

These states are conceptually useful but are not mutually exclusive. Tumors may display partial overlap between immune-excluded, immune-suppressive, and immune-desert features, and may transition between these states over time or under treatment pressure. Accordingly, the table is intended to provide a practical conceptual framework rather than a rigid classification system.

## 3 Mechanistic architecture of resistant niches

Immune exclusion and immune suppression do not arise from a single dominant lesion. In most solid tumors, they emerge from the co-evolution of stromal structure, myeloid instruction, cytokine wiring, vascular dysfunction, and metabolic stress, which together reshape the local tissue context into a niche that limits T-cell access, blunts effector function, and favors adaptive resistance (12, 25). A useful way to think about these resistant niches is not as static histologic categories, but as self-reinforcing ecological states: once stromal remodeling, suppressive cytokines, and dysfunctional innate immune programs become spatially aligned, the TME begins to stabilize its own nonresponsive phenotype (4, 26). This architecture also helps explain why many tumors display partial immune activity yet remain clinically resistant—because immune recognition, trafficking, and killing are being interrupted at multiple levels at once (27).

### 3.1 Stromal barriers: CAF heterogeneity, fibrosis, and matrix remodeling

Among stromal determinants, cancer-associated fibroblasts (CAFs) are arguably the most consistent organizers of resistant niches. Earlier work established that fibroblasts in tumors are biologically active components of the microenvironment that regulate tumor growth, tissue remodeling, and intercellular signaling, laying the foundation for the current view of CAFs as central architects of resistant niches (18, 28). Contemporary studies no longer treat CAFs as a uniform tumor-promoting compartment. Instead, CAFs are increasingly understood as a heterogeneous and plastic population, including myofibroblastic, inflammatory, antigen-presenting, and metabolically specialized states, whose functional significance may vary according to lineage origin, local signaling, spatial position, and tumor context (18, 25, 28, 29). This heterogeneity is not merely descriptive; it is a major source of biological and translational controversy. Although many CAF programs contribute to fibrosis, immune exclusion, angiogenic support, and therapy resistance, not all fibroblast subsets appear uniformly tumor-promoting. Some may retain context-dependent tissue-organizing or tumor-constraining functions (25, 30). This complexity helps explain why indiscriminate stromal depletion has often failed to translate the promise of preclinical models into consistent therapeutic benefit. Accordingly, the field is moving toward precision stromal oncology, in which the goal is not blanket ablation but selective reprogramming of dominant CAF programs that sustain exclusionary or immune-restrictive niches. Nevertheless, the functional interpretation of CAF subsets remains incomplete across tumor types. The stability, plasticity, and therapeutic tractability of specific fibroblast states are still being defined. Some CAF-associated mechanisms can therefore be regarded as well supported, whereas others remain context-dependent or insufficiently validated for broad translational generalization.

The stromal compartment is broader than fibroblasts alone. Pericytes and mesenchymal stromal cells can converge toward CAF-like, pro-angiogenic, immunosuppressive, and pro-invasive phenotypes under the influence of PDGF-B/PDGFRβ, TGF-β, CXCL12, and Notch/ROCK signaling (31). This convergence matters mechanistically because it means that fibrosis, vascular remodeling, and immune dysfunction are not independent layers of the TME; they are frequently produced by interconvertible stromal programs that reinforce each other. In ovarian cancer, for example, CAF–TAM cooperation has been linked to extracellular-matrix remodeling, angiogenesis, cytokine production, and therapy failure, illustrating how stromal heterogeneity is translated into clinically relevant resistance phenotypes (32). In lung cancer as well, recent syntheses emphasize CAF heterogeneity and stromal remodeling as key determinants of immune exclusion, fibrosis, and metastatic seeding (30).

### 3.2 Myeloid dominance: monocytes, TAMs, MDSCs, and suppressive innate circuits

If CAFs build the scaffold of resistant niches, myeloid cells often provide their dominant immunologic logic. Foundational studies on tumor-associated macrophages and myeloid-derived suppressor cells established that these populations are not passive infiltrates, but central regulators of tumor-promoting inflammation, immune suppression, angiogenesis, and metastatic dissemination (33–35). Tumor-resident macrophages, monocyte-derived cells, and MDSCs do not simply accumulate as bystanders; they actively impose suppressive tone through antigen-presentation defects, checkpoint induction, cytokine release, angiogenic remodeling, and effector-cell inhibition (23). This view is strongly supported by earlier work defining MDSCs as systemic and local suppressors of adaptive immunity and TAMs as multifunctional orchestrators of invasion, vascular remodeling, and metastatic progression.

What makes the myeloid compartment especially formidable is its plasticity. At the same time, this plasticity complicates interpretation of the literature. Although suppressive myeloid populations are recurrently linked to immune resistance across tumor types, their phenotypes, ontogeny, and dominant functions are not always consistent across studies, and the relative contribution of TAMs, monocyte-derived cells, and MDSCs may differ substantially by tissue context, treatment history, and analytic platform. Distinct myeloid subsets can assume overlapping suppressive functions despite differing ontogeny, making the TME resilient to single-pathway disruption (23, 26). As a result, many solid tumors maintain a “myeloid-high, T-cell-low-function” state even when checkpoint blockade is pharmacologically adequate. This has made tumor-resident myeloid cells one of the most attractive targets for combination immunotherapy. However, mechanistic centrality does not automatically translate into therapeutic simplicity. Because suppressive myeloid programs are often redundant, adaptive, and spatially intertwined with stromal and cytokine circuits, interventions that appear conceptually compelling in preclinical systems may yield variable results when applied across heterogeneous human tumors.

Importantly, stromal and myeloid programs are not parallel tracks; they are deeply interdependent. CAFs release chemokines and growth factors that favor monocyte recruitment and macrophage polarization, while TAMs in turn sustain fibrosis, matrix remodeling, angiogenesis, and suppressive cytokine release (36). In ovarian cancer, this CAF–TAM partnership is already being recognized as a central resistance axis rather than a secondary feature of tumor progression. In other settings, such as KRAS-mutant tumors, oncogenic signaling amplifies myeloid recruitment and immune suppression by promoting MDSCs, Tregs, and CAF-associated remodeling, thereby linking tumor-intrinsic genotype to suppressive niche formation (24, 37). This crosstalk is one reason why macrophage targeting, chemokine modulation, and stromal reprogramming are increasingly being pursued together rather than as isolated strategies.

### 3.3 Cytokine and chemokine circuits as organizing signals

Resistant niches are also sustained by a soluble regulatory layer, in which cytokines and chemokines act less as individual mediators than as coordinating signals for tissue-level immune behavior. Earlier work on cancer-related inflammation established that cytokine-rich inflammatory networks are integral to tumor development and immune dysregulation, while later studies highlighted STAT3-centered signaling and chemokine-guided immune-cell trafficking as major organizing axes within the tumor microenvironment (38–40). Against this historical background, recent cytokine-centered therapeutic frameworks can be viewed as a translational extension of a longer mechanistic literature rather than an entirely new conceptual starting point. A particularly useful recent framework is the “push-pull” model of cytokine modulation during checkpoint blockade. The “push” component aims to enhance Th1- and cytotoxic-supportive signals, including IL-2, IL-12, IL-15, and type I/II interferons. The “pull” component aims to attenuate dominant suppressive circuits, including TGF-β, IL-6/STAT3, IL-8/CXCR1-2, IL-10, and TNF-driven exhaustion (12, 41). This framing is valuable because it captures an important biological point: resistant TMEs are not maintained simply by the absence of pro-inflammatory signals, but by the active predominance of suppressive cytokine networks that shape spatial architecture, regulate myeloid programs, and induce inhibitory ligands such as PD-L1. This broader view is consistent with earlier work linking inflammatory cytokines, STAT3 activation, and chemokine dysregulation to immune escape and tumor progression (38–40).

These cytokine circuits are not generic across cancers. In head and neck squamous cell carcinoma, recent work highlights recurrent combinations of CAF-driven exclusion, chemokine-dependent myeloid and Treg recruitment, and hypoxia-linked immune dysfunction (4, 42). In lung cancer, IL-6 and TGF-β repeatedly emerge as nodal mediators connecting chronic tissue injury, fibroinflammatory remodeling, and immune suppression (30). In KRAS-driven tumors, altered signaling landscapes similarly shape chemokine output and downstream immune-cell composition, helping explain why co-mutation context can helping explain why co-mutation context can shift tumors toward more immune-inflamed or more immune-poor states (24). Taken together, these findings argue that cytokine biology in resistant niches is best understood as a network property, not a single-target abnormality.

### 3.4 Vascular disorder, hypoxia, and metabolic stress

A further layer of resistance arises from vascular and metabolic misprogramming, which can decouple immune-cell presence from immune-cell effectiveness. Abnormal vessels reduce efficient immune trafficking, create oxygen gradients, and foster nutrient competition, while hypoxia promotes checkpoint upregulation, stemness, and suppressive myeloid conditioning (4, 26, 43). In practical terms, this means that even tumors with measurable lymphocyte infiltration may remain functionally resistant because T cells are operating in a metabolically hostile and spatially fragmented landscape. Recent reviews of urologic cancers and HNSCC converge on this view, emphasizing HIF-linked programs, lactate accumulation, acidification, and nutrient competition as central contributors to T-cell dysfunction and therapeutic failure.

This vascular–metabolic axis is especially clear in chronic inflammatory tumors. In renal cell carcinoma, chronic renal injury and tissue hypoxia appear to sustain NF-κB/STAT3 and HIF-VEGF signaling, generating an “inflammation but constrained” phenotype in which IFN-γ programs, stromal barriers, endothelial inertia, and metabolic stress coexist (43). Similar logic has been proposed in NSCLC, where metabolic reprogramming, immune-cell dysfunction, and physical barriers are integrated into a cold-to-hot transformation framework (44). More broadly, translational reviews increasingly place metabolic targeting, vascular normalization, and stromal disruption alongside checkpoint blockade as co-equal components of TME rewiring rather than optional add-ons (26, 27).

### 3.5 Biomechanical checkpoints: extracellular matrix stiffness as an active suppressor

The extracellular matrix should not be reduced to a passive barrier. Emerging work positions ECM stiffness itself as a biomechanical checkpoint that actively shapes immune exclusion. Stiff matrices alter immune-cell motility, restrict T-cell drop-in, and modify macrophage polarization toward tumor-promoting states, thereby converting a structural abnormality into an immunologic one (11). This is particularly relevant in desmoplastic tumors, where matrix density and rigidity can dominate the functional landscape of the TME. Cholangiocarcinoma and PDAC are illustrative examples: both are characterized by abundant stiff ECM and fibroblast-rich stroma, and both show how matrix remodeling can limit drug access, restrict T-cell penetration, and stabilize suppressive cell-cell interactions (11, 44).

Mechanistically, ECM stiffening also intersects with signaling pathways that amplify resistance. Fibroblast-derived matrix remodeling promotes pro-survival signaling, mechano-transduction, angiogenesis, and immunoregulatory feedback loops, while macrophages both respond to and reinforce the stiffened environment (11, 25). This reciprocity helps explain why purely pharmacologic immunostimulation often fails in highly fibrotic tumors: the problem is not only insufficient activation of effector immunity, but the persistence of a mechanically exclusionary tissue state. Accordingly, strategies that combine ECM modulation or stromal normalization with immune activation are gaining traction across fibrotic cancers (44).

### 3.6 Organ-specific and metastatic microenvironments

Resistant niches are further shaped by the tissue in which they emerge. Organ-specific microenvironments provide distinct stromal templates, vascular behaviors, resident immune populations, and injury-response programs, all of which influence how immune exclusion and immune suppression are expressed. In lung cancer, for instance, recent reviews emphasize not only the primary tumor ecology but also the divergent microenvironments that govern organ-specific metastasis, including brain metastasis (30). In bone-associated tumors such as osteosarcoma, immune exclusion appears to be reinforced by structural and regulatory features of the bone niche, highlighting that exclusion may be especially resilient in tissues with pre-existing physical and immunologic compartmentalization (42). These observations caution against overly generic models of TME resistance and support a more context-aware view in which metastatic site is itself a biologically meaningful determinant of immune state.

**Figure 1:**
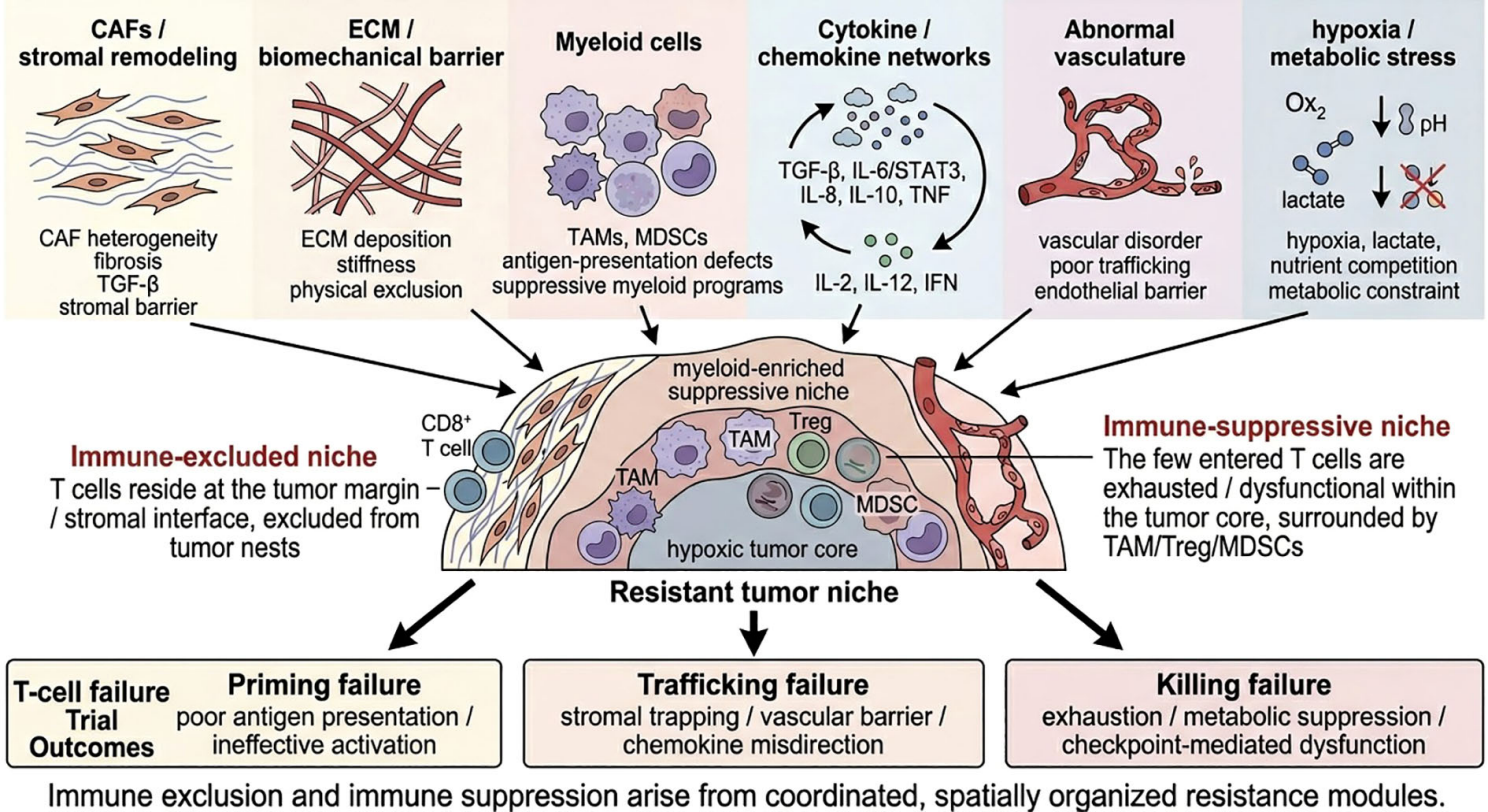
Conceptual architecture of immune-excluded and immune-suppressive tumor niches. Schematic overview of the major resistance modules that cooperatively shape immune-excluded and immune-suppressive tumor microenvironments. Cancer-associated fibroblasts (CAFs), extracellular matrix (ECM) remodeling and stiffening, suppressive myeloid populations, dysregulated cytokine and chemokine networks, abnormal vasculature, and hypoxic/metabolic stress act in concert to establish resistant niches. These interconnected processes restrict effective T-cell priming, impair trafficking and intratumoral infiltration, and weaken cytotoxic function after tumor entry. Immune-excluded niches are characterized by stromal trapping of lymphocytes outside tumor nests, whereas immune-suppressive niches contain infiltrating but functionally restrained immune cells. Together, these features sustain immune escape and limit the efficacy of immune checkpoint blockade.

The clinical implication is straightforward but important: there is unlikely to be a single “master mechanism” of immune-resistant TME across cancers. Instead, resistant niches are assembled from a shared set of modules—stromal remodeling, myeloid dominance, cytokine dysregulation, vascular dysfunction, metabolic stress, and biomechanical exclusion—but the relative weight of each module differs by tumor genotype, tissue context, and treatment history (4, 27, 43). This is precisely why longitudinal and spatial profiling are becoming indispensable. Without resolving how these modules are combined in individual tumors, therapeutic rewiring remains empirical. With such resolution, however, resistant niches can begin to be treated as structured, targetable ecosystems rather than as diffuse background noise (4, 26) (**Figure 1**).

## 4 Spatial biomarkers and emerging technologies for decoding resistant TMEs

**Figure 2:**
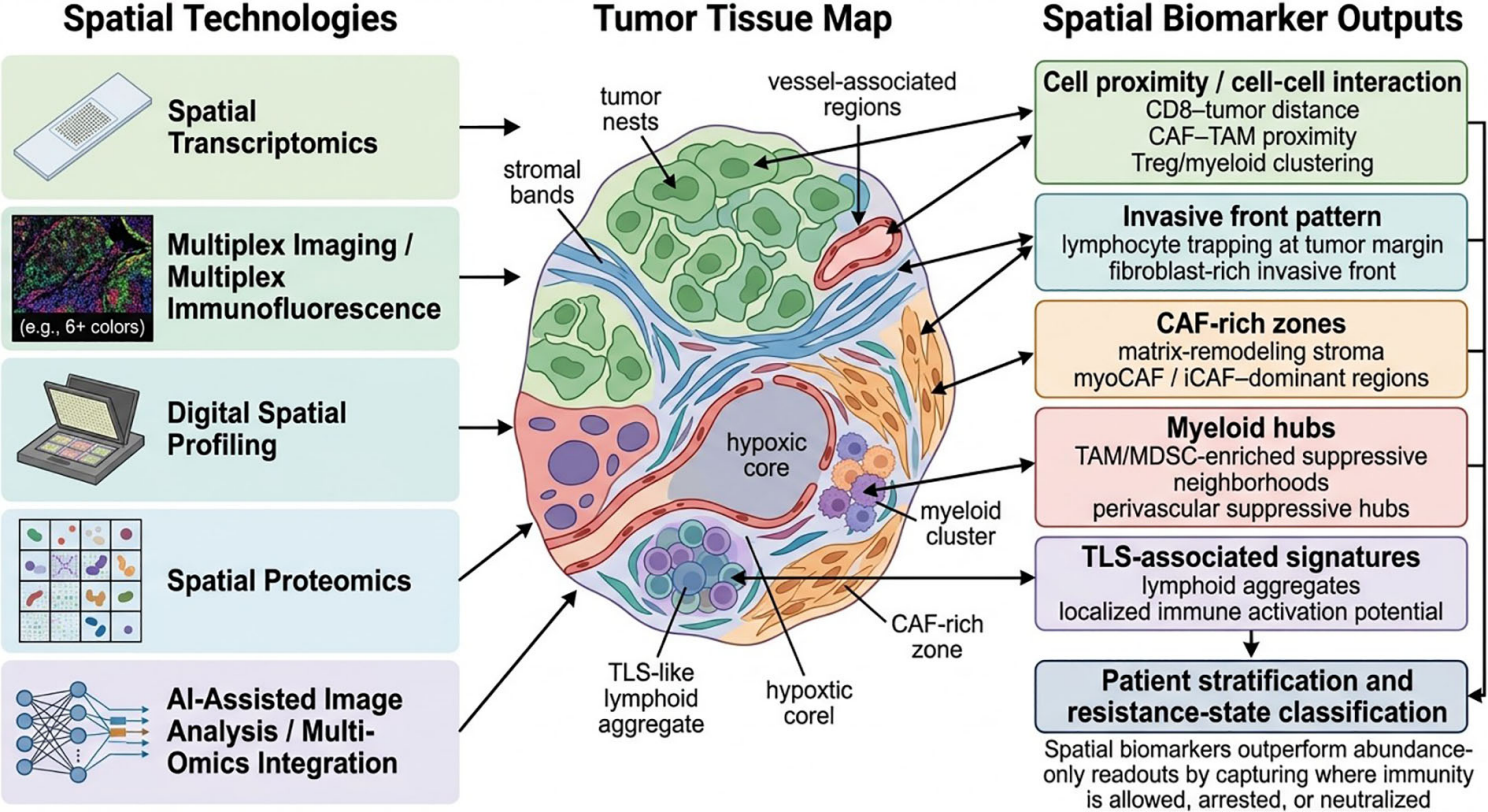
Spatial biomarkers and technologies for decoding resistant tumor microenvironments. Schematic representation of the major spatial technologies used to resolve resistant tumor microenvironments and the corresponding biomarker outputs generated from these platforms. The left panel summarizes representative technologies, including spatial transcriptomics, multiplex imaging, digital spatial profiling, spatial proteomics, and computational multi-omics integration. The central panel illustrates a tumor tissue map containing distinct spatial regions, such as invasive fronts, CAF-rich stromal zones, myeloid-enriched suppressive hubs, tertiary lymphoid structure (TLS)-associated areas, and hypoxic cores. The right panel highlights key spatial biomarker categories derived from these approaches, including cell proximity and interaction patterns, invasive front architecture, CAF-rich zones, myeloid hubs, and TLS-associated signatures. These spatially resolved readouts refine resistance-state classification and improve patient stratification beyond abundance-based biomarkers alone.

The emergence of spatially resolved profiling has changed how resistant tumor microenvironments are interpreted. Earlier bulk and even conventional single-cell approaches were highly informative for defining cell states, but they necessarily stripped away the positional information that determines which cells actually interact, where suppressive circuits accumulate, and how regional microenvironments constrain therapeutic response (7, 45). In the context of immunotherapy, this omission is not trivial. The same tumor may contain activated lymphocytes, suppressive myeloid cells, fibroblasts, and endothelial elements, yet the biological meaning of these populations depends heavily on whether they co-localize, segregate into distinct compartments, or form stable contact networks at the invasive front, in hypoxic cores, or around vessels (7). As a result, the field is moving from cell enumeration toward spatially informed interpretation of resistant TMEs (27). This conceptual shift has an important historical basis. Long before contemporary spatial omics became widely available, landmark studies in colorectal cancer showed that the type, density, and, crucially, the location of immune cells within tumors were strongly associated with clinical outcome, thereby establishing the principle of immune contexture as a clinically meaningful spatial dimension of tumor biology rather than a purely descriptive pathological feature (5, 46, 47) (**Figure 2**).

### 4.1 Why spatial context matters beyond abundance-based biomarkers

Spatial biomarkers are most informative when they distinguish immune-cell location and function, not simply immune-cell quantity. This is also why some of the earliest clinically influential spatial immune biomarkers were not based on high-dimensional omics alone. The development and subsequent international validation of the Immunoscore, which quantifies CD3+ and CD8+ T-cell densities in the tumor core and invasive margin, provided an important proof-of-principle that compartment-aware immune measurement can add clinically relevant prognostic information beyond simple global immune-cell abundance. However, this level of validation should not be generalized to all emerging spatial biomarkers, many of which remain exploratory and lack prospective evidence as predictive tools for immunotherapy selection (48, 49). A tumor with a moderate number of CD8-positive T cells may still behave as immune-excluded if those cells remain stromal or peri-tumoral, whereas another lesion with substantial immune infiltration may remain clinically nonresponsive because the infiltrate is regionally exhausted, metabolically constrained, or counterbalanced by suppressive myeloid-fibroblastic hubs (7). This is especially relevant in cancers with mixed immune phenotypes, where immune-inflamed, immune-excluded, immune-suppressive, and immune-desert regions can coexist within the same specimen or evolve over time under treatment pressure (50). Spatial analysis therefore adds a layer that conventional biomarkers such as PD-L1 expression or total lymphocyte density cannot supply: it reveals where immune activity is allowed, where it is arrested, and where it is neutralized (7, 51).

This point has direct translational relevance. In brain metastases, for instance, the immune microenvironment is often constrained by limited T-cell infiltration and regionally organized cytokine and growth factor signals, making biomarker interpretation highly dependent on tissue context rather than single analytes alone (52). Likewise, lung adenocarcinoma metastases to lymph node, brain, bone, and liver exhibit distinct microenvironmental states despite sharing broad immune-suppressive features, indicating that biomarker interpretation must account for organ-specific spatial ecology as well as cell composition (51). Thus, spatial biomarkers are not simply more detailed biomarkers; they are contextual biomarkers, designed to capture the microregional logic of therapeutic resistance.

### 4.2 Major spatial technologies: strengths, limitations, and clinical positioning

Current spatial platforms differ in resolution, multiplex capacity, analyte coverage, and translational readiness, and these differences shape the kinds of biological questions they can answer. The current platform landscape emerged through a series of methodological milestones. Highly multiplexed imaging approaches, such as multiplexed ion beam imaging and imaging mass cytometry, established high-parameter protein mapping in intact tissues. Iterative fluorescence-based methods, including t-CyCIF and CODEX, further expanded single-cell tissue imaging. In parallel, spatial transcriptomics enabled transcriptome-scale mapping in histological sections, whereas digital spatial profiling extended spatial analysis to region-selective RNA and protein quantification in fixed tissue specimens (53–57). Spatial transcriptomics provides transcript-level mapping while preserving tissue architecture and is particularly useful for discovering regional programs, lineage transitions, and neighborhood-level signaling patterns (7, 45). Multiplex imaging approaches, including highly multiplexed immunofluorescence and mass-imaging platforms, excel at resolving cell-cell proximity and phenotypic organization in intact sections, which is critical when the goal is to identify immune barriers, perivascular niches, or tumor–stroma interfaces (7). Spatial proteomics further strengthens this framework by capturing protein-level states and pathway activity, offering a bridge between mechanistic biology and pathology-oriented biomarker development (58). In melanoma, for example, spatial imaging technologies are already being used to dissect the interplay among tumor cells, stroma, and immune populations in order to inform prognosis and response prediction, although workflow standardization and integration into routine pathology remain important barriers.

A major practical strength of these technologies is that they can be paired with single-cell datasets rather than replacing them. Single-cell RNA sequencing remains powerful for resolving cell states and lineage programs, but spatial transcriptomics restores the missing tissue coordinates needed to interpret how those states are deployed *in vivo* (45, 59). This complementarity is particularly valuable in fibroblast biology. Traditional single-cell studies identified broad CAF heterogeneity, yet newer spatial transcriptomic work shows that CAF subsets such as myofibroblastic, inflammatory, antigen-presenting, matrix-remodeling, and proliferative CAFs are not randomly distributed; rather, they exhibit spatial preferences within tumor cores, hypoxic zones, invasive fronts, and tertiary lymphoid structure-adjacent regions. That shift—from identifying cell states to mapping where those states operate—is precisely what makes spatial technologies so useful for decoding resistant niches (59).

At the same time, spatial methods are not free of limitations. Resolution varies considerably across platforms, coverage is often constrained by panel size or sequencing depth, and analysis pipelines remain sensitive to tissue handling, segmentation strategy, and computational assumptions (7, 58). Importantly, the most biologically interesting tumors are often the most difficult to standardize because necrosis, fibrosis, treatment effect, and tumor-stroma admixture complicate both tissue preservation and signal interpretation. These challenges explain why clinical adoption has lagged behind technical enthusiasm. The core issue is no longer whether spatial biology is informative—it clearly is—but how to define robust workflows that produce reproducible, clinically actionable readouts across laboratories and specimen types. To improve practical readability for translational readers, the major spatial profiling platforms discussed in this section are summarized in **Table 2**, with emphasis on their analyte coverage, strengths, limitations, and representative biomarker outputs.

**Table 2:** Spatial biomarker platforms: strengths, limitations, and representative outputs.

Platform	Main analyte	Strengths	Limitations	Key outputs
Spatial transcriptomics	RNA	Tissue-wide transcriptomic context	Resolution varies	Immune programs; stromal modules; hypoxia; exclusion patterns
scRNA-seq + spatial integration	RNA	Cell states + localization	Integration-dependent	CAF subsets; exhausted T cells; myeloid states; TLS programs
Multiplex IF/t-CyCIF/CODEX	Protein	Single-cell spatial phenotyping	Panel and analysis limits	CD8 exclusion; CAF–T-cell separation; suppressive neighborhoods
IMC/MIBI	Protein	High-dimensional imaging	Specialized, lower throughput	Myeloid hubs; exhausted niches; tumor–immune contacts
Digital spatial profiling	RNA/protein	FFPE-friendly; ROI profiling	ROI-dependent	Tumor/stroma signatures; invasive front; cytokine modules
Spatial proteomics	Protein	Functional pathway readout	Panel constraints	Checkpoints; signaling states; interaction profiles
Spatial metabolomics	Metabolites	Maps metabolic stress	Lower maturity	Hypoxia; lactate-rich regions; metabolic barriers
Computational pathology	Image features	Scalable; clinically accessible	Validation needed	Exclusion scores; stromal metrics; TLS; spatial risk scores

These platforms are complementary. Choice of platform should depend on the biological question, tissue type, required resolution, available specimens, and intended translational application.

### 4.3 Spatial hallmarks of resistant niches

Across tumor types, several recurrent spatial patterns have begun to emerge as hallmarks of immune-resistant microenvironments. One common feature is the immune-excluded rim, in which lymphocytes accumulate at the tumor margin or within stromal corridors but fail to penetrate tumor cell-rich nests (7). A second is the fibroblast-dense invasive front, where matrix-remodeling CAFs, endothelial dysfunction, and myeloid recruitment converge to create a barrier-rich zone associated with recurrence, shortened survival, or diminished immunotherapy responsiveness (59). Third, many tumors contain hypoxic or metabolically stressed cores, which favor myeloid conditioning, effector dysfunction, and altered cytokine landscapes (60). Finally, perivascular and perinecrotic microdomains often act as privileged signaling centers in which gradients of oxygen, stress, and soluble mediators create localized states that are not visible in bulk analyses (60).

These patterns are not merely descriptive. In recent fibroblast-focused spatial syntheses, peri-tumoral enrichment of specific myofibroblastic states has been associated with immune exclusion and shortened survival, while sub-micron interaction networks between CAFs and SPP1-positive macrophages, CXCL13-positive CD8-positive T cells, NK cells, or endothelial cells appear capable of pushing the TME toward either exclusion or activation (59). Similarly, region-specific immune organization has been highlighted in lung cancer and HNSCC, where spatial transcriptomics is beginning to reveal how stromal remodeling, checkpoint programs, and metabolic stress are assembled into spatiotemporally heterogeneous immune landscapes (27, 61). These studies suggest that resistant TMEs are organized less by isolated pathways than by neighborhood-level alliances among stromal, immune, and tumor populations.

Spatial biology has also sharpened understanding of metastatic disease. In brain metastases, biomarker work now emphasizes not only immunologic and proteomic signatures but also the importance of spatially constrained immune dynamics shaped by limited infiltration and local cytokine networks (52). In lung adenocarcinoma metastases, the spatial heterogeneity across lymph node, brain, bone, and liver lesions suggests that immune escape is constructed differently in each organ, even when overlapping biomarkers such as PD-L1, cytokine profiles, or immune-cell ratios are present (51). These observations reinforce an important principle for biomarker development: a marker may be biologically relevant yet still lack predictive value if interpreted without regard to where in the tissue—and in which organ context—it is expressed.

### 4.4 Candidate spatial biomarkers for patient stratification

The most clinically promising spatial biomarkers are unlikely to be single molecules. Historically, one of the clearest proofs-of-principle for this idea came from the evolution of the Immunoscore, which translated the earlier concept of immune contexture into a standardized spatial assay based on immune-cell density in defined tumor compartments. More recent multiplexed imaging studies have extended this logic from compartment-level metrics to cell-neighborhood and interaction-based biomarkers, suggesting that clinically useful spatial signatures will likely combine cell identity, location, and relational architecture rather than rely on single analytes alone (46, 48, 49, 62, 63). Current evidence favors multicomponent spatial signatures that integrate cell state, location, and interaction pattern. These signatures may include T-cell exclusion metrics, CAF distribution, and CAF subtype localization. They may also incorporate the proximity of suppressive myeloid cells to tumor nests, TLS-adjacent immune architecture, regional cytokine-associated states, and combined angiogenesis–stromal–IFN modules (50, 59). Recent reviews on cytokine-guided resistance and renal cancer evolution both converge on the idea that transcriptomic profiles, plasma kinetics, myeloid/neutrophil metrics, and spatial TME readouts may provide complementary information beyond single-parameter tests. However, most candidate spatial biomarkers remain supported by retrospective cohorts, exploratory spatial profiling studies, or biological plausibility rather than prospective treatment-assignment trials. Therefore, spatial biomarkers should currently be viewed primarily as hypothesis-generating and stratification-enabling tools, rather than as clinically validated predictive assays that can independently direct immunotherapy selection (12, 50).

Several specific candidate frameworks are already taking shape. In melanoma, spatial proteomics is being explored to map stromal–immune–tumor relationships with potential relevance to prognosis and therapeutic response (58). In ovarian cancer, spatial transcriptomic analyses of immune-low states are being used to define TAM-rich niches, T-cell exhaustion zones, Treg accumulation, NK-cell dysfunction, and stromal barriers, thereby generating phenotype-guided combination hypotheses (64). In NSCLC, spatial transcriptomics is increasingly positioned not just as a descriptive tool, but as a platform for identifying subtype-specific resistance mechanisms and matching cold-to-hot transformation strategies (27). The unifying message is that spatial biomarkers are most useful when they resolve the dominant resistance module in a given tumor, whether that module is stromal, myeloid, vascular, metabolic, or mixed.

### 4.5 Additional biomarker dimensions: TLSs, neoantigen quality, sex differences, and immune repertoires

Several additional biomarker dimensions deserve attention as complements to spatial TME profiling. Tertiary lymphoid structures (TLSs) may indicate organized local immune activity, including antigen presentation, B-cell maturation, T-cell priming, and coordinated antitumor responses (65). However, their interpretation should consider TLS maturity, location, cellular composition, and relationship to suppressive stromal or myeloid niches, rather than treating TLS presence as a simple binary marker. Tumor mutational burden and neoantigen features provide a tumor-intrinsic dimension of immunogenicity. TMB is useful as a practical surrogate, but neoantigen clonality, HLA presentation, antigen retention, and antigen-presentation competence may be more informative than mutation count alone. Sex-based differences may also influence TME composition and immunotherapy response through sex hormones, sex chromosomes, inflammatory tone, metabolism, and environmental exposures, although these effects remain context dependent and incompletely resolved (65–68). Finally, single-cell TCR and BCR sequencing can complement spatial methods by linking immune-cell location to clonal expansion, antigen-driven immune responses, and regional repertoire sharing. Together, these dimensions reinforce that spatial biomarkers should be interpreted within a broader integrated biomarker framework rather than in isolation.

### 4.6 Integration with engineered and dynamic models

Despite their power, spatial technologies remain observational unless paired with platforms that allow functional testing. This has led to growing interest in 3D and engineered systems that reconstruct microregional heterogeneity, including organoid co-culture systems, microchip-based 3D tumor cultures, and other tissue-engineered approaches capable of modeling zone-specific communication and resistance (60). Even reviews that are not explicitly engineering-focused now point toward organoid models and longitudinal immune snapshots as necessary complements to spatial profiling if biomarker-guided intervention is to become truly predictive (50, 51, 69). The same logic applies to extracellular-vesicle biology, where recent work argues that bulk exosome analysis misses the zone-specific influences exerted by stromal, hypoxic, perivascular, quiescent, and immune-desert regions, and that spatial biology platforms will be essential for resolving these communication networks (60).

**Figure 3:**
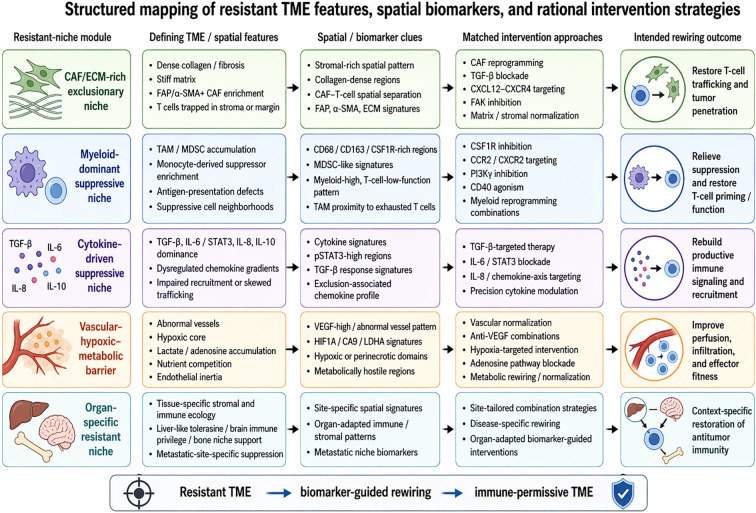
Structured mapping of resistant TME features, spatial biomarkers, and rational intervention strategies. This integrative schematic map dominant resistant-niche modules in immune-excluded and immune-suppressive tumor microenvironments to their defining TME and spatial features, representative biomarker clues, matched intervention strategies, and intended rewiring outcomes. CAF/ECM-rich exclusionary niches are linked to stromal normalization and CAF reprogramming; myeloid-dominant suppressive niches to myeloid-targeting and myeloid reprogramming approaches; cytokine-driven suppressive states to precision cytokine modulation; vascular–hypoxic–metabolic barriers to normalization-oriented interventions; and organ-specific resistant niches to site-tailored therapeutic combinations. The figure emphasizes dominant patterns rather than mutually exclusive states, as many tumors exhibit overlapping resistant modules.

Ultimately, the value of spatial technologies in resistant TME research lies not only in better pictures, but in better decision frameworks. Thus, spatial profiling functions as a bridge between resistant-niche biology and therapeutic prioritization. The resulting mechanism-to-intervention logic is summarized in **Table 3** and **Figure 3**. This makes spatial biology central to the transition from empiric immunotherapy to rational microenvironment-guided intervention. For the purposes of this review, spatial technologies are therefore treated not as a standalone technical topic, but as the bridge connecting mechanistic niche biology to therapeutic rewiring. Taken together, these advances support a more structured translational framework in which dominant resistant-niche modules can be identified through spatial and biomarker-based readouts and linked to mechanism-matched therapeutic rewiring strategies.

**Table 3:** Major resistant-niche modules in immune-excluded and immune-suppressive tumor microenvironments.

Resistance module	Key features	Spatial/biomarker clues	Representative therapeutic rewiring strategies
CAF/stromal remodeling	Fibrosis, ECM deposition, TGF-β-driven exclusion, angiogenic/stromal support	Fibroblast-rich invasive front; stromal T-cell trapping; CAF/ECM signatures	CAF reprogramming, ECM modulation, TGF-β targeting, stromal normalization
Myeloid-dominant immunosuppression	TAM/MDSC accumulation, defective antigen presentation, suppressive cytokines	Myeloid-enriched niches; macrophage-rich stromal zones; myeloid signatures	Myeloid depletion/repolarization, chemokine-axis targeting, macrophage-directed therapy
Cytokine/chemokine dysregulation	TGF-β, IL-6/STAT3, IL-8, IL-10, TNF-associated exhaustion	Cytokine modules; chemokine-misdirected immune positioning; IFN-γ/stromal profiles	Precision cytokine modulation, local cytokine delivery, cytokine-guided ICI combinations
Vascular/hypoxic/metabolic constraint	Abnormal vasculature, hypoxia, lactate accumulation, nutrient competition	Hypoxic cores; perivascular niches; angiogenic/metabolic markers	Vessel normalization, anti-angiogenic therapy, metabolic intervention
Organ-specific metastatic niches	Tissue-specific stromal and immune programs	Site-dependent immune architecture in brain, liver, bone, and lymph node metastases	Site-aware biomarker selection, longitudinal/multi-site sampling, adaptive combinations
TCM-derived adjunctive modulation	Multi-component effects on CAFs, cytokines, checkpoint signaling, and immune-cell function	Immune infiltration change; PD-1/PD-L1-related readouts; TGF-β/stromal programs	CHM-based adjunctive combinations, CAF-directed sensitization, checkpoint-supportive modulation

CAF, cancer-associated fibroblast; CHM, Chinese herbal medicine; ECM, extracellular matrix; ICI, immune checkpoint inhibitor; MDSC, myeloid-derived suppressor cell; TAM, tumor-associated macrophage; TCM, traditional Chinese medicine.

## 5 Therapeutic rewiring of resistant tumor microenvironments

**Figure 4:**
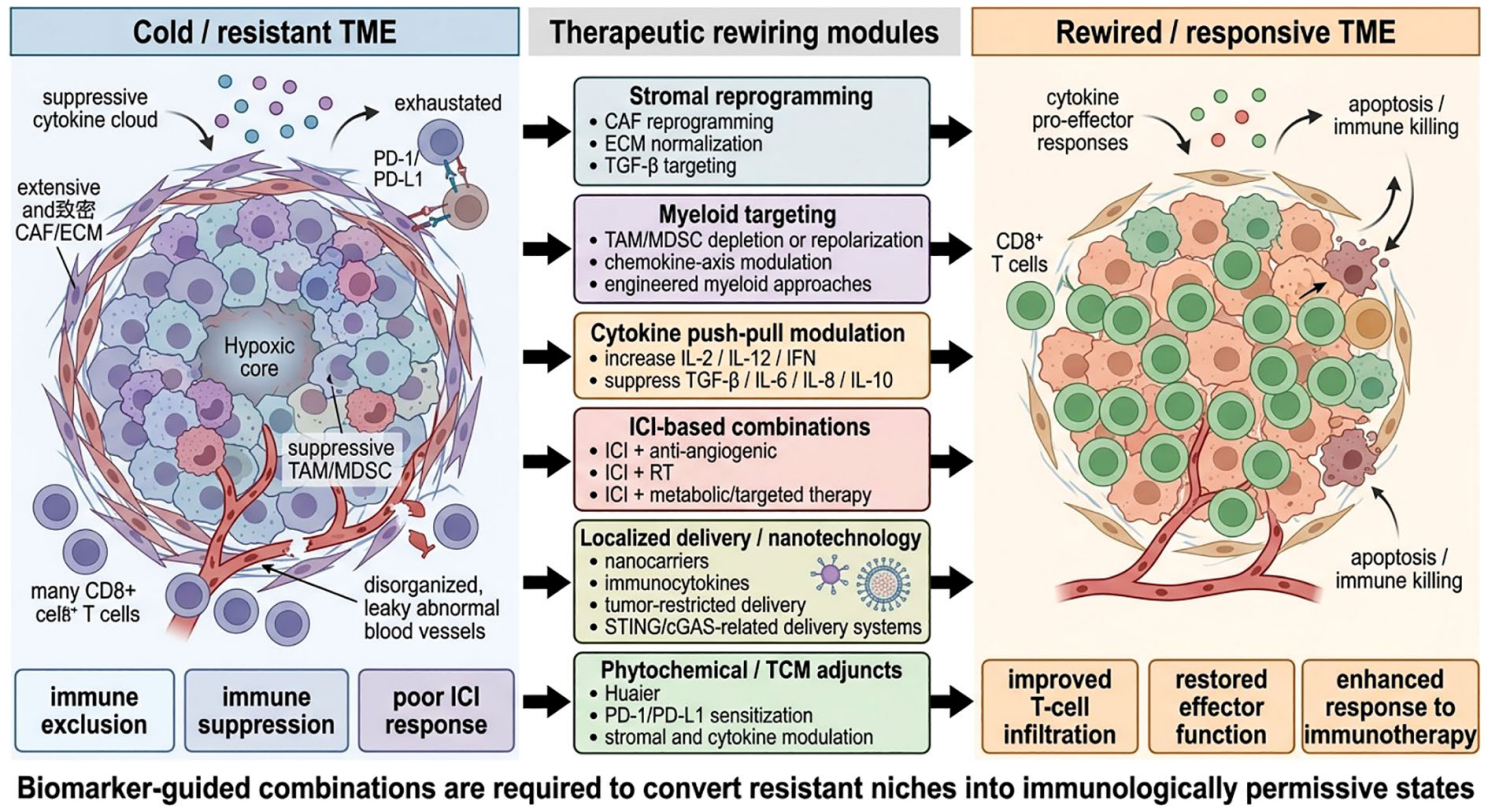
Therapeutic rewiring strategies to convert cold tumors into responsive niches. Schematic summary of the principal strategies for therapeutic rewiring of immune-resistant tumor microenvironments. The left side depicts a cold or resistant tumor microenvironment characterized by stromal exclusion, myeloid-dominant immunosuppression, abnormal vasculature, suppressive cytokine signaling, and limited T-cell infiltration or function. The middle panel outlines six major therapeutic modules: stromal reprogramming, myeloid targeting, cytokine push-pull modulation, immune checkpoint inhibitor (ICI)-based combinations, localized delivery/nanotechnology-enabled intervention, and phytochemical/traditional Chinese medicine (TCM)-derived adjunctive modulation. The right side illustrates the intended rewired state, featuring improved lymphocyte infiltration, reduced suppressive barriers, restored effector-cell activity, and enhanced responsiveness to immunotherapy. The image emphasizes that biomarker-guided combination strategies are required to transform cold tumors into immunologically permissive niches.

If resistant niches are sustained by layered stromal, myeloid, vascular, metabolic, and cytokine barriers, then meaningful clinical improvement is unlikely to come from checkpoint blockade alone. The emerging therapeutic goal is therefore not merely to “stimulate immunity,” but to rewire the tissue context in which immunity operates. In this framework, successful treatment should either remove the barriers that prevent immune-cell entry, restore effector-cell competence within suppressive lesions, or convert nonproductive immune engagement into coordinated antitumor activity (1). Recent translational reviews increasingly converge on this view: conversion of immune-poor or resistant niches into more immune-permissive states is not a single intervention, but a biomarker-guided combination strategy built around the dominant resistance module in a given tumor (1, 4, 12, 26) (**Figure 4**; **Table 4**).

**Table 4:** Dominant barriers, candidate biomarkers, and plausible rewiring strategies.

Dominant barrier	Candidate biomarker	Rewiring priority
CAF/ECM exclusion	CAF/ECM, collagen, TGF-β/CXCL12, stromal T-cell trapping	Stromal normalization; CAF/ECM targeting
Myeloid suppression	TAM/MDSC, CD68/CD163/CSF1R, exhausted T-cell proximity	Myeloid reprogramming; ICI + myeloid targeting
Cytokine dysregulation	TGF-β, IL-6/STAT3, IL-8, IL-10, chemokine misdirection	Precision cytokine/chemokine modulation
Vascular–metabolic barrier	VEGF, HIF1A/CA9/LDHA, lactate/adenosine, hypoxic cores	Vascular normalization; metabolic targeting
Immune-desert/poor priming	Low CD8, weak IFN, poor antigen/DC signals	Immune priming; vaccines/oncolytic/innate activation
Mixed/site-specific resistance	Regional heterogeneity, organ-specific signatures	Spatially guided, adaptive combinations
Tumor-intrinsic immune escape	JAK1/2 loss, B2M/MHC-I loss, antigen loss, WNT/β-catenin, PTEN, STK11/KEAP1	Genomic-guided strategy; restore antigen presentation or IFN-γ responsiveness; target oncogenic immune-evasion pathways; consider MHC-independent immune approaches

This table prioritizes therapeutic logic according to dominant resistance barriers; many tumors contain overlapping modules and may require combination or sequential approaches.

### 5.1 Rewiring stromal and matrix barriers

This strategy is particularly relevant to CAF-rich, fibrotic, and matrix-dense niches characterized by stromal exclusion and impaired lymphocyte access. Among current strategies, stromal reprogramming has become one of the most conceptually mature. The field has moved away from indiscriminate depletion of cancer-associated fibroblasts (CAFs), partly because CAFs are heterogeneous and partly because broad ablation can destabilize tissue architecture in unhelpful ways. This shift is not merely strategic but conceptual. It reflects growing recognition that CAF populations are heterogeneous and that broad stromal depletion may oversimplify fibroblast biology, with the risk of removing tissue elements that are not uniformly tumor-promoting across all contexts. Instead, recent work emphasizes precision stromal oncology, in which CAF subsets are selectively modulated, matrix density is normalized, and fibroblast-driven exclusionary programs are reversed rather than erased wholesale. Vitamin D receptor agonists, retinoids, epigenetic modulators, TGF-β-axis interventions, and ECM-targeting approaches have all been discussed as candidate tools for shifting fibroblast-rich lesions from fibrosis-dominant toward more permissive immune states (25). This logic is especially compelling in desmoplastic tumors such as pancreatic and ovarian cancers, where physical matrix barriers, hypoperfusion, and fibroinflammatory signaling jointly limit immune-cell penetration and drug delivery (21, 70). However, clinical experience has also shown that stromal targeting is not straightforward. In pancreatic cancer, the phase III HALO-301 trial failed to show a survival benefit when pegvorhyaluronidase alfa was added to nab-paclitaxel plus gemcitabine. Early-phase experience with TGF-β-axis inhibition combined with PD-L1 blockade in metastatic pancreatic cancer has also remained modest, despite acceptable tolerability. These findings reinforce the view that stromal intervention is unlikely to succeed as simple matrix depletion or single-axis suppression. Future strategies will require better biological selection, spatial biomarker support, and more precise combination design.

The translational appeal of TME normalization is that it need not act as a standalone therapy. In ovarian cancer, ECM-normalizing strategies are increasingly framed as sensitizers that may improve both chemotherapy and immunotherapy by relieving abnormal interstitial stress, reducing stromal shielding, and facilitating lymphocyte access (70). Similar reasoning applies to pancreatic cancer, where reprogramming rather than indiscriminate destruction of the fibroinflammatory stroma is now being explored as a route to improve the performance of ICIs, cell therapies, and vaccine-based approaches (21, 44). Thus, stromal therapy is increasingly being repositioned from a niche adjunct to a gate-opening intervention that may determine whether subsequent immunologic therapies can function at all (21, 25, 70).

### 5.2 Reprogramming myeloid suppressive circuits

Myeloid reprogramming is most rationally applied to tumors in which suppressive macrophages, monocyte-derived cells, or MDSC-enriched niches constitute a dominant resistance module. A second major therapeutic axis targets the myeloid compartment, which frequently acts as the operational core of immune-resistant TMEs. Current strategies can be grouped into at least four categories: altering the composition of tumor-resident myeloid cells, functionally blocking suppressive myeloid programs, reprogramming these cells toward pro-inflammatory states, and exploiting myeloid cells themselves as therapeutic vehicles (71, 72). Reviews focused on tumor-resident myeloid cells have highlighted targets such as Siglec-15, TREM2, MARCO, LILRB2, CLEVER-1, and chemokine-receptor pathways, while also emphasizing the growing importance of CAR-M concepts, macrophage repolarization, and engineered myeloid delivery systems (73, 74). Importantly, the myeloid targets listed above are not supported by equivalent levels of clinical evidence. Siglec-15, LILRB2/ILT4, TREM2, and CLEVER-1 are in early clinical development, with preliminary safety or disease-control signals but no mature randomized validation. MARCO remains largely preclinical or tissue-based as a therapeutic target. Experience with CSF1R blockade, including the failure of cabiralizumab-based treatment to improve progression-free survival in advanced pancreatic cancer, illustrates that biologically rational macrophage targeting does not necessarily translate into clinical benefit. These findings suggest that target expression alone is insufficient; future myeloid-directed therapy will require biomarker-defined patient selection and evidence that the target marks a dominant suppressive program rather than a bystander macrophage phenotype. The rationale is straightforward: in many myeloid-dominant or immune-suppressive tumors, myeloid cells are both the major suppressors of T-cell function and the most spatially dominant immune populations, making them natural leverage points for rewiring the niche (72). Yet clinical translation has been more difficult than this rationale initially suggested. Myeloid-targeting strategies have shown heterogeneous and sometimes disappointing outcomes, likely because tumor-associated myeloid populations are highly plastic, suppressive pathways are partially redundant, and many trials have lacked sufficiently precise biomarkers to identify patients whose tumors are truly dominated by therapeutically actionable myeloid programs.

This strategy may be particularly valuable where myeloid suppression is deeply entrenched. In head and neck cancer, for example, recent reviews place myeloid recruitment and stromal–myeloid cooperation among the key determinants of ICI failure, supporting combinations that pair PD-(L)1 blockade with VEGFR/multikinase inhibitors, chemokine modulators, or other myeloid-directed approaches (4). In pancreatic cancer, similarly, MDSC-rich and macrophage-dominant niches are increasingly viewed as modifiable drivers of therapeutic refractoriness rather than merely correlates of poor prognosis (21). In addition, not all myeloid-directed strategies are supported by equivalent levels of evidence. Some targets are backed by strong preclinical rationale but limited clinical validation, whereas others remain exploratory and may ultimately require combination-based or biomarker-restricted application rather than broad deployment. More broadly, strategies designed to overcome myeloid-cell-induced immune suppression are attractive because they may not only debulk inhibitory populations but also improve the activity of pre-existing modalities such as checkpoint blockade and adoptive T-cell therapy in immunologically cold tumors (71, 72). Overall, current evidence supports myeloid reprogramming as a promising component of therapeutic rewiring, but not yet as a uniformly mature or generalizable solution across resistant tumor types.

### 5.3 Precision cytokine and chemokine modulation

Precision cytokine modulation is especially relevant when suppressive signaling circuits such as TGF-β, IL-6/STAT3, IL-8, or IL-10 act as major organizers of local immune dysfunction. The most explicit attempt to formalize therapeutic rewiring at the molecular-network level comes from recent work on precision cytokine modulation. Instead of broadly adding inflammatory signals or suppressing one inhibitory cytokine at a time, this framework proposes a “push-pull” strategy: locally amplifying Th1/cytotoxic-supportive signals such as IL-2, IL-12, IL-15, and type I/II interferons while attenuating dominant suppressive circuits including TGF-β, IL-6/STAT3, IL-8/CXCR1/2, IL-10, and TNF-driven exhaustion (12). This approach is attractive because cytokines sit at the intersection of spatial architecture, myeloid programming, checkpoint induction, and effector-cell fitness. As such, they provide a mechanistic bridge between niche biology and treatment design.

However, cytokine therapy has repeatedly shown that biological plausibility does not guarantee clinical utility. Clinical outcomes reinforce that this distinction matters. Anti–PD-L1 plus anti-VEGF strategies have improved outcomes in selected settings, including unresectable hepatocellular carcinoma in IMbrave150 and metastatic nonsquamous NSCLC in IMpower150. However, not all rational vascular–immune combinations reproduce this success. For example, cabozantinib plus atezolizumab in COSMIC-312 did not improve overall survival compared with sorafenib in advanced hepatocellular carcinoma. These divergent results argue against treating combination immunotherapy as a universally transferable template across tumor types (75, 76). Systemic exposure, network redundancy, and compensatory feedback can blunt efficacy and magnify toxicity, which is why newer approaches increasingly emphasize localized or tumor-restricted delivery, including immunocytokines, tumor-activated pro-cytokines, intratumoral gene delivery, and biomarker-guided sequencing with ICIs (12). These principles also resonate with older but still relevant literature on NK–myeloid cytokine crosstalk, which showed that cytokine effects in tumors are highly context dependent and can either restore or further suppress antitumor immunity depending on the cell populations already present (77). Taken together, current evidence supports cytokine modulation not as an isolated therapeutic class, but as a network-tuning strategy that is most likely to work when matched to the dominant spatial and cellular constraints of a given tumor (77).

### 5.4 Combination strategies with checkpoint blockade

Combination strategies with checkpoint blockade are most likely to be effective when selected according to the dominant resistance architecture of the TME rather than applied empirically across biologically distinct tumors. Most resistant solid tumors will likely require combination immunotherapy, but the next generation of combinations should be driven less by empirical stacking and more by the biology of the resistant niche. Recent reviews across HNSCC, lung cancer, urologic malignancies, and broader translational oncology consistently highlight several convergent pairings: ICIs with anti-angiogenics or vessel-normalizing agents; ICIs with TGF-β/CAF-directed therapies; ICIs with myeloid or chemokine modulators; and ICIs with metabolic interventions designed to relieve lactate accumulation, acidification, or nutrient competition (4, 26, 43, 78). This is an important conceptual shift. The question is no longer simply whether PD-1 or PD-L1 blockade should be intensified, but which microenvironmental barrier should be dismantled to make checkpoint therapy meaningful. Clinical outcomes reinforce that this distinction matters. Anti–PD-L1 plus anti-VEGF strategies have improved outcomes in selected settings, including unresectable hepatocellular carcinoma in IMbrave150 and metastatic nonsquamous NSCLC in IMpower150; however, not all rational vascular–immune combinations reproduce this success, as cabozantinib plus atezolizumab in COSMIC-312 did not improve overall survival versus sorafenib in advanced hepatocellular carcinoma. These divergent results argue against treating combination immunotherapy as a universally transferable template across tumor types (79, 80).

Radiotherapy remains one of the most established partners for this approach because it can increase antigen release, augment T-cell priming, and reshape the irradiated TME in ways that favor systemic immunity. Recent reviews emphasize that RT–ICI combinations may generate abscopal effects and durable immune activation when properly sequenced, although these benefits remain highly contingent on local stromal context and treatment-induced suppressive rebound (81). Similarly, immunogenic cell death (ICD) strategies—including ferroptosis, necroptosis, and pyroptosis induction—have gained attention because they may help convert cold tumors into inflamed lesions by increasing tumor antigenicity and promoting lymphocyte influx. In SCLC, where conventional ICIs benefit only a minority of patients, both ICD-based combinations and noncanonical immunomodulatory strategies are being investigated as ways to overcome intrinsic exclusionary or macrophage-centered suppressive states (43, 82).

Tumor-intrinsic targeted therapy may also contribute to rewiring when it disrupts the molecular drivers of immune suppression. KRAS-directed approaches are a good example. Beyond their direct antitumor effects, KRAS G12C inhibitors have been discussed as potential TME-restoring agents that may improve checkpoint responsiveness, especially when combined with additional pathway inhibitors such as SHP2-targeted drugs (83). This line of thinking is important because it integrates oncogenic signaling with microenvironmental intervention, rather than treating them as separate therapeutic domains. In practical terms, the most effective future regimens may be those that simultaneously reduce tumor-cell fitness and dismantle the suppressive niche that protects the tumor from immune attack (26, 83).

### 5.5 Nanotechnology, local delivery, and engineered therapeutic platforms

A particularly active frontier in therapeutic rewiring is the use of nanotechnology and engineered delivery systems to localize microenvironmental intervention. This strategy is appealing because many TME-directed drugs fail not for lack of biological relevance, but because systemic administration cannot achieve adequate activity within resistant niches without unacceptable toxicity (12). Nanotechnology-based platforms offer one solution by enabling selective delivery of immunomodulators, cytokines, ICD inducers, or metabolic agents to the tumor site, thereby widening the therapeutic window and increasing local immune activation (26, 84). Reviews in ovarian cancer, breast cancer, and nanomedicine-focused oncology increasingly treat nanotechnology not simply as a delivery tool, but as a means of programming the TME itself, whether by restoring T-cell and NK-cell function, reducing suppressive cell populations, or remodeling stromal and hypoxic barriers (26, 85, 86).

More specialized platforms are also emerging. Engineered myeloid cells have been proposed as cellular carriers capable of homing to tumors and delivering therapeutic payloads while simultaneously reshaping macrophage-dominant niches (71). Biohybrid nanoplatforms, iron oxide nanoparticle systems, and related theranostic constructs have been reviewed as ways to bridge localized tumor killing with systemic immune priming, although the extent to which these approaches will translate clinically remains uncertain (87). At a conceptual level, these innovations all reflect the same shift: away from thinking of the TME as an obstacle that passively reduces efficacy, and toward treating it as a targetable tissue compartment that can be directly manipulated through delivery design (71, 87, 88).

### 5.6 TME rewiring in specific resistant tumor contexts

Although the broad principles of rewiring are shared across cancers, the therapeutic emphasis differs by disease context. PDAC remains the canonical example of a fibroinflammatory, immune-poor tumor that frequently displays immune-excluded and immune-suppressive features in which CAF targeting, metabolic reprogramming, vaccine-based priming, KRAS-directed therapy, and combinatorial immunotherapy are all being pursued as means of overcoming profound stromal and myeloid resistance (21, 44, 89). This designation is supported by earlier mechanistic studies showing that the desmoplastic stroma of PDAC creates a major physical barrier to effective drug delivery, while fibroblast-derived chemokine signaling can actively mediate immune evasion and limit productive antitumor immunity (Provenzano et al., 2012; Feig et al., 2013). Importantly, subsequent work also showed that indiscriminate depletion of carcinoma-associated fibroblasts may aggravate immunosuppression and worsen disease behavior, reinforcing the current shift from stromal ablation toward selective stromal reprogramming (90). In lung cancer, the range of proposed TME-directed strategies is especially broad, spanning immune microenvironment modulation, metabolic and epigenetic intervention, nanotechnology-based delivery, and biomarker-guided integration with targeted therapy or radiotherapy (78). In urologic cancers, by contrast, recent reviews place particular emphasis on anti-angiogenic combinations, ECM-modulating agents, hypoxia-targeted drugs, and stratification approaches informed by single-cell and spatial profiling (43). These differences are instructive: the best rewiring strategy is unlikely to be universal, but it may still be built from a common therapeutic vocabulary.

### 5.7 TCM-derived adjunctive strategies for therapeutic rewiring of the TME

Traditional Chinese medicine (TCM), particularly Chinese herbal medicine (CHM), is best positioned in this review as an adjunctive and hypothesis-generating TME-modulating strategy rather than as a stand-alone anticancer therapy (91). Its relevance to mechanism-based TME rewiring lies in the fact that some CHM-derived interventions may act on multiple resistance layers simultaneously, including CAF activity, TGF-β-related stromal signaling, checkpoint-associated immune dysfunction, cytokine balance, and immune-cell infiltration. In this sense, CHM may be conceptually aligned with the broader theme of multi-compartment TME modulation, especially when used as a sensitizing partner for standard therapies rather than as a replacement for them (92, 93).

A representative example is Huaier, which has recently been shown to enhance immunotherapeutic sensitivity in triple-negative breast cancer by suppressing cancer-associated fibroblasts and attenuating TGF-β/SMAD signaling (94). For example, Huaier has been reported to enhance immunotherapeutic sensitivity in triple-negative breast cancer by suppressing cancer-associated fibroblasts and attenuating TGF-β/SMAD signaling, thereby shifting the immune landscape toward a more immune-infiltrated and functionally permissive phenotype. Other formula-based CHM approaches have also been discussed in relation to CAF-related remodeling, extracellular-matrix regulation, VEGF-associated angiogenesis, and PD-1/PD-L1-related immune modulation (93, 95). However, the current evidence remains heterogeneous, with important limitations related to herbal composition, dose standardization, mechanistic attribution, and prospective clinical validation. Therefore, TCM/CHM-derived approaches should be interpreted as adjunctive, biomarker-dependent, and still clinically immature components of TME rewiring, with future value likely to depend on standardized preparations, spatial or molecular biomarkers, and rigorously designed combination studies (92, 96, 97).

## 6 Clinical translation: biomarkers, trial design, and unresolved challenges

A central lesson from recent TME research is that mechanistic insight alone does not guarantee clinical impact. Many resistant niches can now be described in considerable molecular detail, yet translation remains slow because the clinical problem is not simply identifying one more suppressive pathway; it is deciding which barrier is dominant in which patient, at which disease stage, and at which time point during therapy (26). This is why the field is gradually moving away from static, one-dimensional biomarker models and toward integrated frameworks that combine stromal, myeloid, angiogenic, metabolic, and spatial information (50, 51). In practical terms, the translational challenge is no longer whether the TME matters, but how to convert its complexity into tractable clinical decision rules.

### 6.1 Why many TME-targeting strategies fail in translation

One reason many TME-directed strategies underperform clinically is that resistant niches are redundant by design. Blocking a single suppressive pathway may produce only transient benefit because parallel circuits can compensate. This is especially likely when stromal remodeling, myeloid dominance, checkpoint induction, and metabolic stress coexist in the same lesion. A second reason is that not all biologically plausible targets are supported by equally mature evidence. In several areas of TME research, strong mechanistic rationale has preceded reliable clinical validation. This creates a gap between conceptual attractiveness and therapeutic reproducibility. Apparently conflicting findings may also emerge across studies because tumor type, sampling region, treatment exposure, and biomarker definition differ substantially. The same pathway may therefore appear dominant in one cohort but secondary in another (12, 43). Such complexity also helps explain why apparently conflicting findings may emerge across studies: depending on tumor type, sampling region, treatment exposure, and biomarker definition, the same pathway may appear dominant in one cohort yet secondary in another. This is especially evident in tumors that remain “inflammation but constrained,” where measurable immune activation coexists with endothelial inertia, CAF-rich stroma, and metabolic stress, thereby blunting the effect of immunotherapy intensification (50). In addition, TMB should be interpreted together with neoantigen quality, clonality, HLA presentation, and antigen-presentation competence, because mutation count alone may not capture whether tumor antigens are retained, visible, and immunologically productive.

A second obstacle is spatial heterogeneity, both within tumors and across metastatic sites. Even when a biomarker appears promising in one tissue region, it may not represent the dominant microenvironmental state elsewhere in the same tumor or in anatomically distinct metastases (51, 52). In lung adenocarcinoma metastases, for example, lymph node, brain, bone, and liver lesions share broad immune suppression but differ markedly in immune composition, metabolic dependencies, and local stromal or tissue-specific constraints, which has obvious implications for treatment response and biomarker interpretation (51). Brain metastases provide a related example: limited immune infiltration, cytokine- and growth factor–dependent immune dynamics, and the anatomical constraints of the brain microenvironment complicate direct extrapolation from extracranial biomarker models (52). These data strongly suggest that translational failure often reflects sampling mismatch as much as biological complexity.

A third and often underappreciated challenge is that many candidate biomarkers are evaluated as if they were stable, when in reality resistant TMEs are dynamic ecosystems that change during treatment. Recent work in renal cell carcinoma is particularly instructive here: immuno-inflammatory, immuno-excluded, and immune-desert states may coexist and transition over time, meaning that a baseline biomarker can rapidly lose relevance once therapy reshapes angiogenesis, cytokine tone, stromal structure, or immune composition (50). Similar concerns are raised across translational reviews that argue for repeated “immune snapshots” rather than reliance on pretreatment tissue alone (26, 43, 50). This temporal dimension is likely one reason why otherwise plausible combinations fail in later-stage studies despite encouraging early biomarker associations.

### 6.2 From single biomarkers to integrated biomarker systems

For clinical translation, the main challenge is not whether single biomarkers are imperfect, but how to combine complementary readouts into usable decision frameworks. PD-L1 expression, tumor mutational burden, lymphocyte density, cytokine profiles, myeloid metrics, and spatial TME features each capture different aspects of resistance. Their value is likely greatest when interpreted together to identify the dominant resistance program in a given tumor (26, 51, 52).

Recent reviews already outline the kinds of composite biomarkers that may prove most useful. These include IFN-γ-related features interpreted alongside angiogenesis and stromal modules, plasma cytokine dynamics integrated with myeloid or neutrophil metrics, and spatial indicators that distinguish peri-tumoral trapping from true intratumoral immune penetration (12, 50). In renal cancer, integrative biomarkers that couple IFN-γ characteristics with angiogenic, stromal, and spatial modules have been proposed as more predictive than isolated tests. In metastatic lung adenocarcinoma, PD-L1, cytokine profiles, immune-cell ratios, and metabolic markers have been highlighted as complementary rather than competing indicators (51). Collectively, these frameworks support a shift from “Which biomarker is best?” to “Which biomarker combination best identifies the dominant resistance program?”

### 6.3 The translational value of spatial and multi-omics profiling

In practice, spatial and multi-omics profiling may first serve as trial-enabling tools before becoming routine clinical diagnostics. Spatial transcriptomics, spatial proteomics, multiplex imaging, and related platforms allow clinicians and investigators to distinguish immune-rich but ineffective lesions from truly immune-desert states, while also identifying fibroblast-rich fronts, perivascular suppressive hubs, or spatially confined macrophage/Treg programs that may not be evident in bulk datasets (52, 58, 64). This matters because treatment selection depends not only on whether antitumor immunity is weak, but on the specific resistant state involved—immune exclusion, myeloid-dominant suppression, exhausted infiltration, immune desertification, or mixed regional states (64, 78). By resolving these configurations, spatial biomarkers can inform whether a patient is more likely to benefit from stromal normalization, cytokine modulation, myeloid targeting, or intensified checkpoint-based combinations.

At the same time, spatial technologies face real translational hurdles. Workflow standardization, tissue handling, computational reproducibility, image analysis pipelines, assay cost, and integration into routine pathology remain unresolved problems. Melanoma-focused spatial proteomics reviews make this point clearly: the biological utility of spatial imaging is increasingly evident, but turning it into a clinically robust platform still requires refinement of methods and adaptation to pathology workflows (58). Thus, although spatial profiling is likely to become central to precision immuno-oncology, its near-term role may be as a trial-enabling and stratification technology before it becomes a routine frontline diagnostic.

### 6.4 Designing smarter trials for TME-guided therapy

If biomarker interpretation must become more nuanced, clinical trial design must evolve in parallel. Traditional trial structures that enroll patients based on tumor type and then layer TME-directed therapies onto standard ICIs risk obscuring benefit because they do not distinguish between patients whose dominant resistance programs are stromal, myeloid, vascular, or metabolic (12, 26). A more rational design would use biomarker-matched modular trials, in which patients are assigned to add-on strategies according to defined resistance signatures rather than broad histology alone (50). For example, a tumor with strong stromal/angiogenic modules may be better suited to vessel normalization or CAF-targeted combinations, whereas a lesion dominated by cytokine/myeloid suppression may require different add-on logic (12, 43, 50).

Future TME-guided trials should incorporate several design principles. First, biomarker assessment should not be limited to pretreatment tissue. Longitudinal biopsies, circulating immune measurements, and on-treatment profiling may help identify immune-state transitions and emerging resistance (50). Second, trial endpoints should extend beyond response rate and progression-free survival. They should also evaluate whether the intended niche rewiring has occurred. Relevant pharmacodynamic endpoints may include changes in stromal architecture, myeloid composition, cytokine kinetics, or spatial immune penetration (12). Third, metastatic site should be considered in trial design. The dominant resistance program may differ between brain, liver, bone, and lymph node metastases. These differences may influence both biomarker interpretation and treatment response. In short, TME-informed trials will likely need to be adaptive, longitudinal, and compartment-aware rather than purely histology-driven (51, 52). In short, TME-informed trials will likely need to be adaptive, longitudinal, and compartment-aware rather than purely histology-driven.

### 6.5 Unresolved challenges: standardization, feasibility, and evidence hierarchy

Despite substantial progress, several unresolved issues continue to limit clinical implementation. One is assay standardization. Multi-omics and spatial pipelines differ in tissue requirements, computational assumptions, and analytical thresholds, making cross-study comparison difficult (58). Another is clinical feasibility: deep profiling is not always practical in routine oncology, especially for patients with small biopsies, necrotic lesions, or anatomically difficult metastatic sites (51, 52). There is also a persistent evidence hierarchy problem. Many promising biomarker models remain supported primarily by retrospective associations, exploratory cohorts, or mechanistic inference rather than prospective validation (26, 43). Until these biomarkers are embedded into interventional studies, their true predictive value will remain uncertain. In additions, a further reason for translational failure is that some tumors classified as TME-resistant may in fact be dominated by tumor-intrinsic immune escape. For example, loss of antigen presentation, defective IFN-γ/JAK–STAT signaling, or oncogene-driven immune exclusion may render checkpoint intensification or stromal/myeloid rewiring insufficient. In such cases, the correct therapeutic question is not only how to recondition the TME, but whether tumor cells remain recognizable and susceptible to immune effector mechanisms. This distinction argues for integrating TME profiling with tumor genomic, antigen-presentation, and IFN-γ-response biomarkers in future trials.

A further challenge is balancing precision with practicality. The field now has the conceptual tools to distinguish immune-excluded, immune-suppressive, mixed, and transitioning states, but not every clinic can deploy spatial transcriptomics, repeated tissue sampling, and full multi-omics integration for every patient. The most useful translational frameworks may therefore be those that derive scalable clinical surrogates from deeper biology—for example, simplified transcriptomic panels, blood-based cytokine metrics, digital pathology scores, or reduced spatial signatures that preserve biological relevance while improving real-world feasibility (12, 58). This will be crucial if TME-guided therapy is to move beyond specialist centers.

### 6.6 A practical translational roadmap

Taken together, the current literature supports a practical roadmap for clinical translation. First, resistant TMEs should be classified using integrated biomarker logic, not single-marker shortcuts (12, 26, 50, 51). Second, spatial and longitudinal profiling should be used to determine whether a tumor is primarily excluded, myeloid-dominant, cytokine-driven, angiogenic, metabolically constrained, or mixed (50, 64). Third, add-on therapies should be selected to match the dominant resistance program rather than applied uniformly (26, 43). Finally, trials should be designed to test not only whether patients benefit, but whether the intended niche rewiring actually occurs (12). This is likely the most important conceptual shift in the field: from treating the TME as background biology to treating it as a measurable, targetable, and monitorable clinical state.

## 7 Conclusions and future perspectives

Immune-excluded and immune-suppressive tumor microenvironments should no longer be regarded as vague background features of solid tumors. They are now better understood as structured, dynamic, and spatially organized resistance states in which stromal barriers, myeloid dominance, cytokine networks, vascular dysfunction, and metabolic stress converge to interrupt the cancer-immunity cycle at multiple levels (1, 27). This conceptual shift has important consequences for the field. It suggests that the next major advance in immuno-oncology will not come from escalating checkpoint blockade in a uniform manner, but from identifying the dominant resistance architecture in each tumor and intervening accordingly (12, 27). In that sense, the future of TME-directed therapy lies less in discovering a single universally effective agent and more in learning how to classify resistant niches with sufficient precision to support rational therapeutic rewiring (1, 51).

A major priority moving forward is to replace oversimplified binary labels such as “hot” and “cold” with spatially and temporally resolved immune-state models. Accordingly, we use “cold” only as a broad umbrella descriptor and reserve “immune-excluded,” “immune-suppressive,” and “immune-desert” for more specific spatial or functional states. This effort will also require greater discipline in distinguishing mechanisms that are broadly reproducible from those that remain context-dependent, tumor-specific, or still primarily hypothesis-generating. Recent work in metastatic lung adenocarcinoma, brain metastases, and ovarian cancer already shows that poor response to immunotherapy may arise from distinct and only partially overlapping states, including immune desertification, stromal exclusion, regional T-cell exhaustion, NK-cell dysfunction, and TAM-rich suppressive hubs (51, 52, 64, 98). These states are not interchangeable, and they are unlikely to respond to identical combinations. Future classification systems will therefore need to distinguish not only whether a tumor is immunologically constrained, but also how and where that constraint is organized within tissue space and metastatic context. Such a framework would provide a stronger biological basis for selecting stromal normalization, myeloid targeting, cytokine modulation, or cell-based strategies in a more personalized manner.

A second priority is the deeper integration of single-cell, spatial, and longitudinal profiling into both discovery and clinical development. Spatial transcriptomics and related approaches have already demonstrated their value in resolving immune-cell positioning, regional stromal programs, and treatment-associated shifts in microenvironmental organization (45). However, their greatest future contribution may be in enabling dynamic biomarker systems rather than static baseline classification. By combining spatial profiling with serial sampling, liquid biopsies, cytokine kinetics, and digital pathology, future studies may be able to monitor whether a tumor is genuinely transitioning from an excluded or suppressed state toward a productive immune state during treatment (45, 52). This will be essential if TME-targeted combinations are to move from descriptive biology to adaptive clinical decision-making.

A third direction concerns functional validation. Although spatial and multi-omics technologies have greatly improved our ability to map resistant niches, they remain largely observational unless paired with experimental systems capable of testing causality and therapeutic vulnerability. The future therefore lies not only in better profiling, but also in better models—particularly patient-derived organoids, engineered co-culture systems, and other platforms that can reconstruct stromal architecture, immune exclusion, and microregional heterogeneity with higher fidelity (51). This is especially relevant for metastatic disease, where organ-specific niches in the brain, liver, bone, or lymph node may each impose different constraints on immune-cell trafficking and effector function (51, 52). More predictive functional models could help determine which resistance programs are reversible, which are compensatory, and which require parallel targeting from the outset (45, 51).

Therapeutically, the most promising future strategies are likely to be matched combinations that address complementary layers of resistance simultaneously. Cold-to-hot transformation frameworks in NSCLC, cytokine-guided modulation strategies, immunogenic cell-death induction, and engineered myeloid or innate immune approaches all point toward the same conclusion: successful therapy will probably require coordinated intervention at the level of priming, trafficking, and local execution rather than intensification of one axis alone (12, 27, 71, 82). The precise combination may vary by tumor type and spatial phenotype, but the broader principle is already clear. Tumors dominated by fibroinflammatory exclusion may require stromal normalization before T-cell–directed therapy can work, whereas lesions dominated by suppressive cytokine or myeloid programs may benefit more from local network reprogramming or engineered innate-cell strategies. Future clinical trials should therefore be designed not only to test efficacy, but also to verify whether the intended niche rewiring actually occurred (45).

Finally, the field should remain cautious but ambitious. The growing literature on immune-resistant TMEs has made it clear that no single biomarker, no single technology, and no single therapeutic class will fully solve the problem of immunotherapy resistance in solid tumors (1, 58). Yet this complexity should not be read as a reason for therapeutic pessimism. On the contrary, it suggests that progress will come from integrating biological detail into a more disciplined translational framework: one that combines spatially informed classification, biomarker-guided treatment matching, dynamic monitoring, and mechanism-based combination design (1, 27, 58). If these pieces can be aligned, the study of immune-excluded and immune-suppressive tumor microenvironments may shift from descriptive taxonomy to a genuinely actionable model of precision oncology.
